# How to Characterize the Function of a Brain Region

**DOI:** 10.1016/j.tics.2018.01.010

**Published:** 2018-02-28

**Authors:** Sarah Genon, Andrew Reid, Robert Langner, Katrin Amunts, Simon B. Eickhoff

**Affiliations:** 1 Institute of Neuroscience and Medicine (INM-1, INM-7), Research Centre Jülich, Jülich, Germany; 2 Institute of Systems Neuroscience, Medical Faculty, Heinrich Heine University Düsseldorf, Düsseldorf, Germany; 3 School of Psychology, University of Nottingham, Nottingham, UK; 4 C. and O. Vogt Institute for Brain Research, Heinrich Heine University Düsseldorf, Düsseldorf, Germany

## Abstract

Many brain regions have been defined, but a comprehensive formalization of each region’s function in relation to human behavior is still lacking. Current knowledge comes from various fields, which have diverse conceptions of ‘functions’. We briefly review these fields and outline how the heterogeneity of associations could be harnessed to disclose the computational function of any region. Aggregating activation data from neuroimaging studies allows us to characterize the functional engagement of a region across a range of experimental conditions. Furthermore, large-sample data can disclose covariation between brain region features and ecological behavioral phenotyping. Combining these two approaches opens a new perspective to determine the behavioral associations of a brain region, and hence its function and broader role within large-scale functional networks.

## What Does Any Part of the Brain Do?

Ever since humans have scientifically investigated the mind, understanding how it is organized at the level of its biological substrate (i.e., the brain) has remained challenging. For over a century, great progress has been made in mapping the human brain (based on various characteristics), leading to a rapidly expanding number of parcellation schemes and atlases detailing the organization of cortical areas and modules [1]. Several studies have demonstrated that the structural segregation of the cerebral cortex into different areas (distinguishable based on their biological properties, such as molecular, cellular, or fiber architecture [2,3]) is closely related to its functional segregation [4] and, in turn, its organization into functional networks [5].

Current conceptualizations of brain function as a Bayesian machine, in which brain areas are seen as connected and relatively specialized computational units, are in contrast with the actual available knowledge about functional specialization. Studies over the past century show that the understanding of brain–behavior relationships has been an interdisciplinary endeavor, resulting in rich and heterogeneous patterns of behavioral functions for many brain regions. After reviewing the most common approaches that have contributed to this endeavor, we propose that assessing the relative functional specialization of brain regions requires a critical change in viewpoint, wherein the *a priori* defined construct is the brain region and the unknowns are the behavioral functions associated with it. In that perspective, recent advances in data aggregation offer novel opportunities for a systematic characterization of brain regions across a range of behavioral conditions and phenotypical features. Such an integrative approach could bring us to a pivotal stage in the history of brain mapping and cognitive neuroscience, in which we lift the conceptual fog that has clouded structure–function relationships in the brain, and focus on future formal conceptualizations of functional segregation and integration.

## Brain Areas as Connected Computational Units

The first theories regarding functional specialization of brain areas (which later led to the concept of functional segregation) had already been proposed in the early 19th century by Gall (whose view was later referred to as ‘phrenology’) [6]. However, many ‘functions’ that were associated with certain parts of the outer skull would not be considered as functions from a modern point of view (with the exception of language). The following decades were enlivened by debates on localizationism versus connectionism (for a detailed review, see [7]). The pioneering work performed by Paul Broca and Carl Wernicke in the 19th century evidenced specific behavioral impairment following focal brain lesions, but, at the same time, it was also realized that the attribution of a specific function to a cortical area is related to its anatomical connectivity with distant brain regions. This was illustrated by Wernicke, who introduced the first network view for language comprehension and production [8].

Following this view, the concept of disconnection syndromes refuted strict localizationism as a complete or sufficient account of cortical organization [7]. Accordingly, the human brain mapping field currently relies on the assumption that the brain is governed by two fundamental principles of functional organization: segregation and integration [7,9,10]. The former refers to the fact that the cerebral cortex is not a homogeneous entity but can be subdivided into regionally distinct modules (cortical areas or subcortical nuclei), based on functional and structural properties [11,12]. The latter emphasizes that no brain region is by itself sufficient to perform a particular cognitive, sensory, or motor function. Rather, all mental capacities rely on a dynamic interplay and exchange of information between different regions [13,14].

Importantly, these principles (functional segregation and integration) do not contradict each other, since integration can be conceptualized as interaction between relatively specialized regions, each subserving a distinct process [9,15]. Accordingly, each area can perform a limited range of functions, but the concrete behavioral output depends on which inputs have been processed (from afferent connectivity) and which signal is sent to which other areas (based on efferent connectivity). In this ‘intrinsic’ and ‘connectivity’-based functional specialization of brain areas (developed in [16]), these latter can be conceptualized as relatively specialized computational units, the observed behavioral effects of which depend on the coactivity (and thus information sent and received) of other areas.

Considering brain areas as computational units raises the question of the mechanism of the computation, or basically the question of ‘what does the brain do and how?’ A relatively well-acknowledged view addressing this question is that the brain works as a Bayesian machine [17], computing probabilities that minimize uncertainty [18] and support decision making [19]. This view has been successful, for instance, in explaining perceptual processes as integrative processing of probabilistic distributions [20]. It has also been adapted to explain higher aspects of cognition, such as human optimistic bias [21] and, relatedly, the role of the lateral prefrontal cortex in updating beliefs [22].

Importantly, this ‘Bayesian brain’ view entails an important shift in the conceptualization of ‘functions’. Traditionally, assigning functions to brain regions has mainly been based on conceptualizations of functions from many different disciplines that are interested in the study of the mind and behavior. Here we use the term ‘behavioral function’ to refer to these primarily psychology-related constructs. ‘Episodic memory’, ‘working memory’, ‘motor preparation’, ‘visual attention’, ‘memory consolidation’, ‘speech production’, ‘perspective taking’, and ‘emotional regulation’ are a few examples of these behavioral functions. However, the Bayesian brain hypothesis entails a different conceptualization of the functional specialization of brain regions. Specifically, ‘function’ refers to a computational operation performed by a given region, which contributes to the observed behavioral output. To incorporate this viewpoint, we use the term ‘operation-function’.

In the following sections, we first illustrate the heterogeneity of behavioral functions that have been assigned to brain regions, according to previous approaches; these approaches are then reviewed. We then consider how functional specialization from such conceptualizations might be used in conjunction with recent advances in data aggregation methods to search for the core operation-function of brain regions.

## Functional Specializations as Polyhedra

For any brain region, we can think of many different behavioral functions, based on the perspective from which we consider this brain region. In practice, most of these behavioral functions can somehow be related to one another and seem to comprise a core computational function (i.e., an operation-function) that grounds all behavioral associations but remains latent and is not directly observed. In other words, our current knowledge of the functional specialization of a given brain region can be conceptualized as a polyhedron with its many sides (i.e., many behavioral functions), the sum of which can only be appreciated by investigation from many different perspectives, but whose core center remains intangible.

This conceptual polyhedron can be illustrated by one of the most studied parts of the brain, the hippocampus. It has been associated with different memory functions, such as episodic [23], autobiographical [24], explicit [25], contextual [26], or associative [27] memory, and also with several ‘processes’, including declarative [28] or incremental learning [29], recollection [30], encoding [31], retention [32], consolidation [33], novelty detection [34], binding [35], comparator [36], mismatch detection [37], pattern separation [38], and inferential processes [39]. Furthermore, the hippocampus has been associated with particular ‘behavioral domains’ and ‘tasks’ such as spatial navigation [40], spatial discrimination [41], scene imagination [42], prospection [43], and allocentric representation [44]. Finally, it has been assumed that the hippocampus supports more complex behavioral constructs, such as creative thinking or flexible cognition [45].

How has such a ‘functional polyhedron’ been created? Actually, the hippocampus, like many brain regions, is a complex structure that can be engaged in many different behavioral functions, according to the context and to its interaction with other brain regions. Accordingly, different fields have used different approaches and conceptual frameworks to infer brain–behavior relationships, capturing one of its many behavioral associations. That is, ascribing behavioral functions to a brain region has been a multidisciplinary endeavor, resulting in multiple ontologies (Box 1) and different levels of description of mental functions, ranging from high-level behavioral descriptions to individual tasks and isolated processes hypothesized by cognitive models [46]. Generally, when these constructs have been related to the brain, the discipline has shaped the ontologies, and the inferential approach has driven the level of description, producing heterogeneous conceptual associations for any brain region. In the following sections, we review the inferential approaches used in those different disciplines, and their concepts, advantages, and drawbacks.

## How Have Functions of Brain Regions Been Inferred?

### The Lesion-Deficit Approach

One of the first approaches linking brain and behavior was the lesion-deficit approach. Following the pioneering work of Broca and Wernicke in the 19th century, one of the most famous examples in the 20th century was the study of patient H.M. by Brenda Milner and colleagues. This patient had medial temporal lobe resection (mainly the hippocampus), which resulted in severe anterograde memory deficits [47]. This led to the inference that the hippocampus plays a crucial role in the acquisition of new memories. As exemplified in this famous study, a ‘behavioral function’ is thus inferred from an observed ‘dysfunction’ (here anterograde amnesia) following restricted brain damage or loss. The main strength of this approach is the causal nature of the relationship between the two studied variables (brain and behavior). That is, observing a dysfunction following damage to a specific brain region allows one to infer a crucial role of this region in the respective behavioral function within the intact brain. This strength goes with the **epistemological** (see Glossary) limitation of being only quasi-experimental, as an experimental approach supporting causality implies the ability to demonstrate that alternative explanations have been eliminated. Specifically, in the lesion-dysfunction approach, the effect of prelesion factors (e.g., subclinical strokes and/or cognitive impairment) cannot be ruled out. In other words, the behavioral deficit (i.e., the effect on the dependent variable) could (partly) be driven by factors other than the lesion *per se*, thereby threatening **internal validity** [48]. Furthermore, at the brain level, several issues arising from the spatially structured distribution of lesions [49] and the influence of neuroplasticity (functional and structural adaptation to damage) can limit the inferential power of lesion-deficit mapping (Box 2). Despite these limitations, this approach has shaped many of the most pre-eminent assumptions about functional specialization, and is still considered a benchmark (due to its causal mechanism) against which findings obtained from other approaches are discussed [49].

### The Stimulation Approach

A more recent, experimental approach, mirroring the lesion-deficit approach but applied to healthy participants, is the study of behavioral consequences of virtual lesions created with brain stimulation techniques. In particular, brain activity can be locally impaired with **transcranial magnetic stimulation (TMS), transcranial direct current stimulation**, or transcranial alternating current stimulation. Likewise, the opposite effect (i.e., facilitation by increasing cortical excitability to enhance behavioral performance) is also possible, depending on the protocol [50,51]. These approaches test cause–effect relationships by experimentally manipulating local brain activity and examining its effects on behavior [52]. While representing a powerful experimental approach, internal validity can also be limited by the influence of individual cortical geometry and the relative lack of focality, as well as by the limited range of regions that can be targeted (Box 2). At the behavioral level, in contrast to the lesion-deficit approach, stimulation approaches do not tap into everyday behavior in natural settings. For the sake of experimental control and constraints of the laboratory setting needed for stimulus delivery, behavioral functions are usually operationalized from cognitive models (e.g., ‘memory recall’ [53]), and inference is made on isolated behavioral parameters such as reaction time. Thus, compared with the lesion-deficit approach, experimental brain stimulation can offer higher internal validity, but has limited **ecological validity**.

### The Activation Approach

In recent decades, neuroimaging techniques such as **positron emission tomography (PET)** and **fMRI** have produced a rapid growth in the study of brain–behavior relationships [54], by revealing localizations of brain activity changes induced by mental operations. fMRI quickly became preferred over PET for mapping task-evoked activity because it has a better spatial and temporal resolution [55]; and can hence localize activity changes during specific mental events, such as successful memory encoding [56]. With this technological progress, cognitive psychologists gained a new tool to test and refine cognitive models and theories [57]. In this particular framework, the activation approach can be considered as experimental because it allows the researcher to freely manipulate an independent variable (behavioral condition) and observe its effect on the dependent variable (brain activation). For example, fMRI can provide support for dual-process cognitive models (such as recollection versus familiarity) by demonstrating that the two processes evoke distinct patterns of activity. However, addressing such questions with fMRI requires well-controlled designs, using two conditions which differ only in respect to the target processes (i.e., a ‘pure insertion’; cf. [58]). For example, isolating ‘recollection’ in an fMRI scanner could be operationalized by contrasting recognition of intact word pairs (which engage both recollection and familiarity) with recognition of recombined word pairs (driven only by familiarity) [59,60]. Hence, mental functions are studied using very precise and therefore restricted experimental implementations inferred from cognitive theories. Consequently, as in stimulation studies, the mental operations a participant engages in are conceptually distant from everyday functions (such as the vivid recollection of a recent meeting), therefore limiting ecological validity. With regard to internal validity, the assumption of pure insertion (the assumption that extra processes can be inserted purely, without changing existing processes or eliciting new processes), upon which the interpretation of activation relies, has been questioned (cf. [58]). For example, the internal validity of an fMRI study can be questioned by the fact that epiphenomena of the experimental setting (such as higher attentional demands in a task than in a control condition) cannot easily be dissociated from task-specific effects.

### The Degeneracy Principle

Historically, lesion-deficit approaches (be they neuropsychological or stimulation based) were generally considered to be important with respect to the study of functional specialization of brain regions, because observing a relationship between a focal lesion and a behavioral deficit suggests that this region is necessary for performance. Hence, the lesion approach was long considered the gold standard for identifying the necessity of a brain region for a given function. By contrast, the activation approach was considered the optimal approach for identifying which brain areas were sufficient for a given behavioral function. Accordingly, it was initially hoped that a combination of lesion-deficit mapping and activation approaches would identify the necessary and sufficient brain regions for a given behavioral task [58]. However, this view had to be revised subject to the degeneracy principle (i.e., the fact that one unique behavioral output or outcome can be achieved by different neurocognitive systems [61]). This degeneracy theory resulted in a conceptual mourning in the human brain mapping field, as it was now considered that ‘there may be no necessary and sufficient brain area’ for any behavioral function [58].

### The Structure-Behavior Correlation Approach

In behavioral science, an alternative to the experimental approach for probing associations between variables in natural conditions is to examine the relationships among naturally occurring variations in different variables in a correlative manner [52]. In the study of brain–behavior relationships, the correlational approach can be used to relate neurobiological and behavioral characteristics through their covariation across individuals [62]. Using **MRI**, this is most commonly performed by testing the correlation between brain morphology (such as local gray-matter volume or cortical thickness [62]) and behavioral measures across a group of individuals [63]. Structural brain–behavior correlations include studies on age, gender, and genetic differences (e.g. [64]), studies on cognitive abilities and other psychological features derived from tests or questionnaires (e.g., personality traits [65]), as well as studies aiming to identify morphological correlates of specific clinical symptoms measured with clinical ratings scales. This approach thus allows a conception of ‘behavioral function’, comprising complex phenotypes such as skills or clinical symptoms that are evident in everyday life. For example, episodic memory is frequently probed with the California Verbal Learning Test [66], which has been inspired by the real-life situation of learning a grocery list, hence probing progressive acquisition and consolidation of information in memory (e.g., [67]). Contrasting stimulation and activation studies, but mirroring the lesion-deficit approach, ‘behavioral function’ is thus not an abstract, experimentally controlled process, but a more ecological quantification of everyday cognition. Nevertheless, the inferential power (i.e., internal validity) of the correlation approach is undermined by a lack of experimental control, which implies that possible alternative processes and strategies may yield the same or similar behavioral outcomes [61]. The internal validity is further reduced by the consideration that behavioral measurements could be relatively noisy proxies of the latent construct(s) they aim to target [68]. This concern is especially noteworthy for scores based on subjective reports, the reliability of which is frequently questioned [68]. Moreover, neurobiological features like local volume or cortical thickness estimates are influenced by numerous possible factors, which may show complex relationships with the covariate of interest. This, in turn, may lead to spurious brain–behavior associations (cf. discussion [69]).

### Summary and Conclusions

In summary, associations between behavioral functions and brain regions have been studied by different research fields, which have different concepts of behavioral functions and use different inference approaches. Beyond technical strengths and weaknesses, the potential of these different approaches to associate particular behavioral functions with particular brain regions has been discussed with respect to ecological validity and internal validity (two epistemological qualities considered in behavioral sciences). With all their different strengths, these approaches have together contributed to assign multiple behavioral functions, corresponding to different levels of description, to brain regions.

Although the complementarity of the different approaches can be seen as offering richness in behavioral associations for brain regions, the original goal of individual studies was typically to focus on a behavioral function and map it to a brain region, rather than elucidating the exact function of a given region. That is, the *a priori* defined construct was a mental operation, and the object of inference was the brain region that was related to it. We would contend that this *modus operandi* has only a very limited capacity to answer the initial question: ‘What does any part of the brain do?’ In particular, any inference about the role of any brain region that is derived using this *modus operandi* is complicated by the principle of degeneracy [61].

For example, investigation into the association between the hippocampus and associative memory retrieval can be obscured by the fact that recalling an association of two items can be performed either by retrieving a unitized item integrating both components, or by retrieving the two associated items; with the second cognitive strategy being more likely to recruit the hippocampus [70,71]. Accordingly, regardless of the approach used, finding a role for the hippocampus in the behavioral outcome depends on the neurocognitive aspects that the behavioral paradigm or measure mostly captures, and the neurocognitive system that the individual(s) recruit (different participants can recruit different neurocognitive systems). Consequently, identifying a role of the hippocampus in associative retrieval could require several studies, covering a very heterogeneous range of behavioral paradigms or measures and performed across different population samples.

In conclusion, assessing the relative functional specialization of brain regions critically requires a change in viewpoint, where the *a priori* defined construct is the brain region and the unknowns are the behavioral functions associated with it [72]. This implies screening a vast range of potential behavioral associations for a given brain region, and examining which of these are associated with the region of interest in an unbiased, statistically testable manner that accommodates the aforementioned complementarity of different approaches with respect to behavioral aspects. Recent advances in both data availability and statistical methods have provided very promising avenues for such region-of-interest-based approach.

## Recent Tools Aiding Progress

### Activation Data Aggregation

An overview of the behavioral functions engaging a given brain region could be achieved by scanning a group of individuals for a large range of behavioral conditions that target different mental functions. This approach has recently been undertaken with 12 participants as part of the Individual Brain Charting project.^i^ With its ongoing development of specific decoding tools, this and related projects could significantly contribute to our understanding of the behavioral engagement of brain regions, providing rich and heterogeneous patterns of region–behavior associations at the individual level. Nevertheless, ensuring that patterns of association go beyond the idiosyncrasies of the specific experimental designs and participants will require data integration across many independent studies [73]. Thus, a ‘subject level’ functional polyhedron that capitalizes on aggregation of experiments within-subject could complement findings from across-studies data aggregation.

Integration across studies has now become possible due to two initiatives compiling the results of published activation studies: BrainMap [74,75] and Neurosynth [76]. Although differing in their approach to data extraction and labeling (Box 2), both contain the coordinates of local activation maxima as reported by many thousands of neuroimaging papers, along with descriptions of the behavioral conditions that yielded the respective activity patterns (e.g., ‘saccades’). By applying statistical tests accounting for the base rate of activation for a given region and the base rate of each behavioral condition in the database, the consistency of particular behavioral associations across thousands of studies can be analyzed for any region of interest. Such approaches could be seen as ‘functional behavioral profiling’ (‘functional’ referring to the use of activation data).

**Figure 1.**
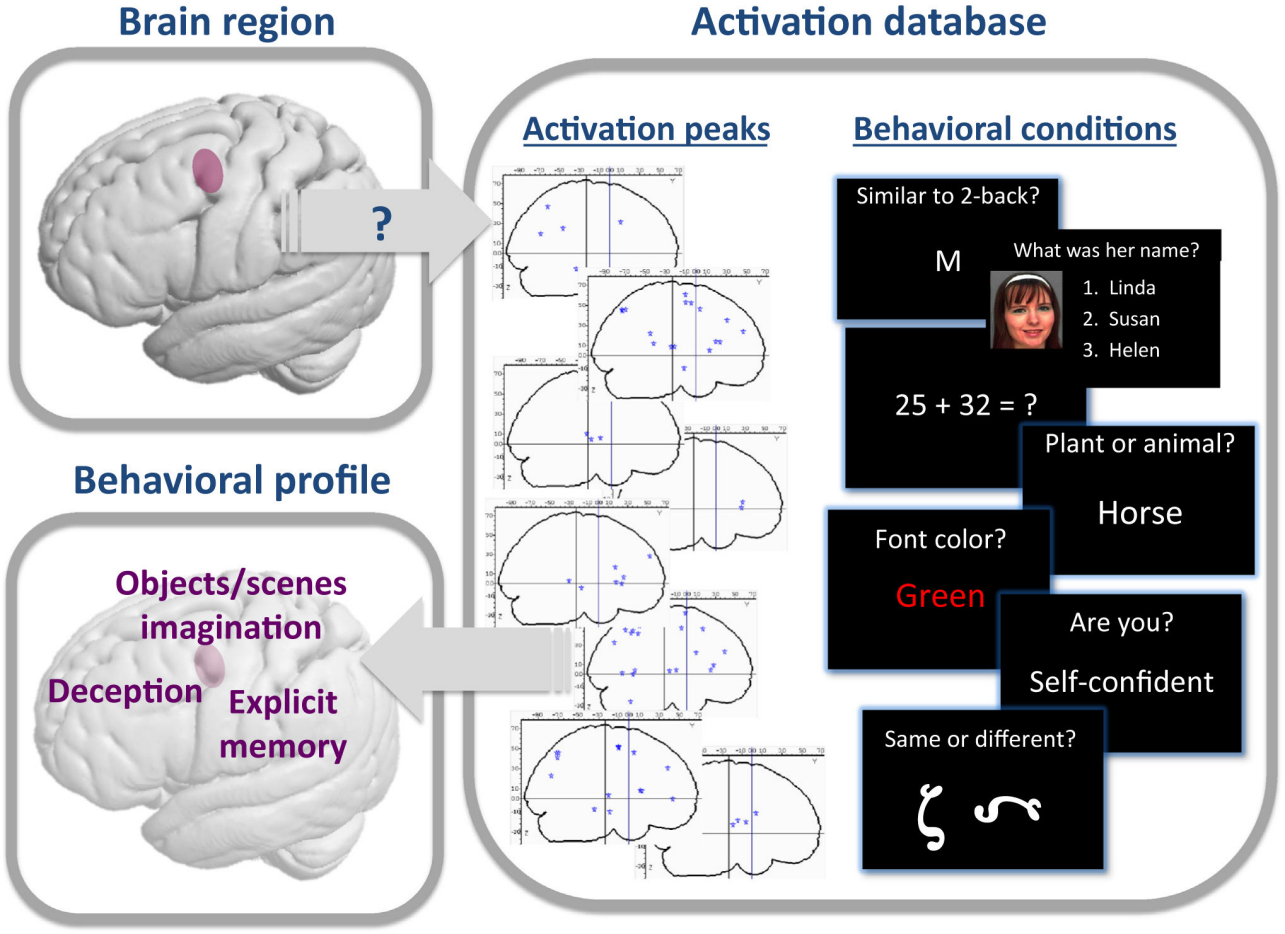
Illustration of Behavioral Functional Profiling for the Left Rostral Dorsal Premotor Cortex (PMd). Activation databases (such as BrainMap and Neurosynth) contain a collection of activation peaks that have been reported in stereotactic space in scientific papers, as well as information on behavioral conditions associated with these peaks (based on the behavioral task that the participants had to perform in the MRI scanner). For a given brain region-of-interest (here the rostral left PMd), we searched among all the peaks of activation reported in the BrainMap database for those that were located in this region. In this database, the behavioral condition related to each peak is specified in terms of behavioral paradigms and behavior domains. Examining the behavioral paradigms and behavioral domains in which the peaks of activation were consistently reported in the region-of-interest allowed us to establish a behavioral profile of this region. As illustrated in the left inferior panel, the left rostral PMd was found to be activated in experimental tasks probing explicit memory, object or scene imagination, and deception [78]. The face used to illustrate a recognition paradigm comes from the Glasgow Unfamiliar Face Database (GUFD) [104].

Using such an approach, it has recently been demonstrated that the anterior insula is engaged in a very wide range of fMRI tasks [77], suggesting a generic functional role, such as task engagement maintenance; that could account for all the more specific mental processes that have previously been discussed for this region. As illustrated in this example, the patterns of associations across a wide range of tasks can foster new hypotheses, approximating as much as possible the core role of the region (and thus its operation-function), beyond the behavioral **ontology** of the original studies or the database. In a recent study, screening the range of studies activating the left rostral dorsal premotor cortex (PMd) revealed that this subregion was activated whenever the task required abstraction from the actual spatial (e.g., scene imagination), temporal (e.g., explicit memory), or mental frame (e.g., deception) ([78], Figure 1). This observation was only possible after integrating activations across different tasks and behavioral domains, and we can speculate that this ‘abstraction’ property actually reflects the use of sequential processing (spatial or temporal) in the PMd for various types of predictions beyond the current framework, in line with the Bayesian brain hypothesis.

Although databases of activation data have existed for many years, systematic ‘functional behavioral profiling’ using these databases is still in its infancy. While this approach shows great potential for disclosing wide patterns of associations, many statistical considerations have to be taken into account. This difficulty has been illustrated recently by a vigorous debate over the functional specialization of the anterior cingulate cortex (ACC), based on conflicting conclusions derived by two groups of authors [79-81]. Such discussions highlight the need to critically investigate the inferential approaches that rely on different statistical considerations, when aiming to comprehensively characterize the pattern of associations for a given brain region. Somewhat relatedly, the use of activation databases for behavioral profiling of brain regions has, to date, focused on single databases while, as discussed in Box 3, BrainMap and Neurosynth show complementary limitations and advantages, suggesting that their combination could provide a more comprehensive profiling and better overview than the previous focus on either of them in isolation. Finally, while a quantitative summary of activation data may thus disclose patterns across tasks from different research fields, it does not allow for disentangling whether the engagement of the brain region plays a crucial role in task performance or whether it is just an epiphenomenon related to experimental implementation (such as more intense visual fixation or cognitive engagement). This highlights the potential benefit of a correlational approach using more naturalistic tasks to complement our view on behavioral associations for a given brain region. The potential outcomes and limitations of such an approach are discussed in the next section.

### Correlation in Large-Scale Population Samples

**Figure 2.**
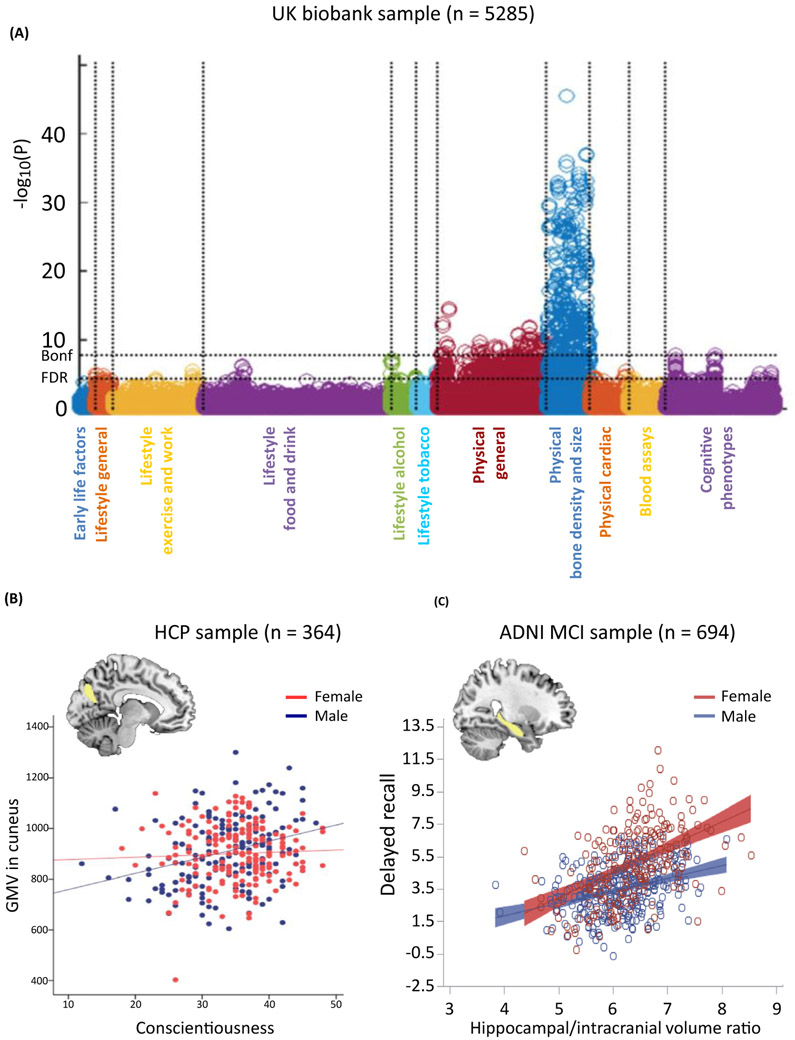
Structural Brain–Behavior Correlation Approach. (A) Manhattan plot relating a set of cerebral measures derived from anatomical images to non-brain phenotypical variables (1100 variables clustered into major groups along the x-axis) in the UK BioBank cohort. For each variable, the significance of the cross-subject correlation with each brain measure set is plotted vertically in units of −log 10 [86]. (B) Positive correlation between conscientiousness scores and gray-matter volume (GMV) in the cuneus in males only, within a sample from the Human Connectome Project (HCP) [65]. (C) Significant relationship between hippocampal volume and immediate recall performance at the Rey Auditory Verbal Learning Test interacting with gender in a sample of participants with mild cognitive impairment (MCI) from the Alzheimer’s Disease Neuroimaging Initiative (ADNI) [87]. Abbreviations: Bonf, Bonferroni correction; FDR, false discovery rate; *n*, number of participants.

An emerging ethos of data sharing has promoted open access to a growing number of large datasets of neuroimaging and phenotypical data [82,83]. The Human Connectome Project (HCP, [84]), the 1000Brains study [85], and the UK Biobank [86] are instances of such initiatives. They provide multimodal brain imaging, information on history and current life-style, questionnaire scores, and a substantial range of standard neuropsychological measures that address several cognitive dimensions, such as working memory, executive functions, and verbal learning. Brain characteristics measured with MRI in large-scale population data show a natural covariance with cognitive phenotypes (Figure 2A, [86]), which allows a standard correlation approach for identification of specific brain regions that are related to behavioral dimensions of *a priori* interest (such as conscientiousness [65], Figure 2B). It would also allow evaluation of a specific association between a region of interest and *a priori* selected behavioral variables (such as hippocampus volume and memory performance [87], Figure 2C) within a hypothesis-driven framework.

Supporting the validity of such an approach to capture brain–behavior relationships, measures tapping into similar aspects of behavior tend to show correlation in the same brain region (such as immediate recall and delayed recall in a list-learning task [87], or extraversion and conscientiousness in the assessment of personality [65]). Such relationships open the perspective of an exploratory approach searching for significant associations between brain measurements in an *a priori* selected region of interest and a wide range of psychometric variables. That is, capitalizing on the hypothesis that neurobiological features such as gray-matter volume and cortical thickness covary locally with behavioral characteristics [62,88], the behavioral functions in which a given brain region potentially play a relative role could be inferred from its structural brain–behavior correlation across the range of phenotypical variables. As this approach is built on a very distinct conceptualization of mental functions through the assessment of complex, ecologically more valid tasks than the specific contrasts offered by the activation approach, it should reveal complementary patterns of behavioral association for any given region. Thus, ultimately, for a given brain region, the pattern of behavioral associations revealed by this structural correlation approach should be integrated with the pattern of behavioral associations derived from activation data, to offer a multiconceptual and multimodal pattern of behavioral associations for any brain region. This hybrid approach would, in turn, help to develop new hypotheses on the operation-function of any region.

## Toward Testing of Interaction Models and Finer Scales

The correlation approach and, more generally, any data-driven approach using big datasets in which researchers just ‘let the data speak’ have their own limitations, as the neuroimaging and psychometric data may contain substantial noise [89], with confounding factors partly driving the observed effect [68]. Ultimately, the function of any brain region should be considered within an integrative approach, including not only patterns revealed by local properties, but also interactions with other brain regions. In other words, data-driven approaches that adopt a functional segregation view and are applied to aggregated observations, offer a great opportunity for exploratory work and discovery science, but any resulting ‘operation-function’ hypothesis should be integrated into a functional model, tested with a hypothesis-based approach. As discussed previously, each approach has its own technical and scientific strengths and limitations, suggesting that a comprehensive evaluation of a given hypothesis should capitalize on a combination of different approaches, rather than focus exclusively on any one of them (see Outstanding Questions). Technical advances can now offer better experimental control, such as by combining electroencephalography (EEG) with focal brain stimulation [90]. Furthermore, Bayesian-based methodological frameworks, such as dynamic causal modeling, have been successful in recent years in the statistical testing of neurocognitive models, and can now even be extended to combined EEG–fMRI paradigms [91]. Altogether, these technical and methodological developments hold great promise for testing models of operation-functions computed by different brain areas in interaction.

As previously discussed [16], in humans, assigning behavioral functions to neuronal populations using a noninvasive neuroimaging approach is restricted by the spatial resolution and precision of these techniques. In particular, individual differences in neuroanatomy can result in mixed functional activation patterns when data from several participants are aggregated in fMRI studies. Several improved approaches for areal alignment across participants in MRI data have been proposed, allowing further examination at the individual level of the structure–function relationships that are suggested by large-scale activation data aggregation (Box 4). Nevertheless, the spatial scale of local functional specialization remains limited by the intrinsic spatial resolution of MRI. That is, activation/structure–behavior covariance within a predefined area could represent a mixture of spatially proximate but functionally independent units, whose separation cannot be resolved by MRI. One consequence of this intrinsic spatial resolution is that inference approaches can only assign a ‘supraordinal’ function to a given brain area, summarizing the different functions performed by different neuronal subpopulations contained within a voxel. Invasive human studies and animal models could further test this hypothesis and help to refine our knowledge of the functional specialization of particular cell populations, such as place cells in the hippocampus (see e.g., Stachenfeld *et al*. [92]), and thus complement functional specialization patterns derived from other approaches.

## Concluding Remarks

Despite centuries of study of brain–behavior relationships, a clear formalization of the function of many brain regions, accounting for the engagement of the region in different behavioral functions, is lacking. Recent progress in data aggregation methods has opened a wide avenue for a systematic, multiconceptual characterization of behavioral associations for any brain region. On the one hand, previous decades of fMRI and PET activation experiments have provided a wealth of results that can be used to shift the perspective toward searching for the range of behavioral associations of brain regions using a quantitative approach. On the other hand, large-scale population datasets open new possibilities for a complementary approach based on covariance between neurobiological and behavioral features. This hybrid behavioral profiling could leverage the myriad of behavioral aspects of any brain region, hence progressively unveiling the nature of the function of brain regions and networks.

## Box 1. Matching Brain Organization and Cognitive Ontologies

One important issue in the study of brain–behavior relationships is whether currently available ontologies and taxonomies can be mapped onto the brain. That is, if we could capture the exact topographical organization of the brain, it seems unlikely that we would find concepts at the current level of description of behavior that could be mapped to the identified biological units. It seems likely that the behavioral structure as it is implemented (i.e., ‘coded’) in the brain does not fit with past and contemporary cognitive theories of behavioral processes.

As reviewed in [16], cognitive scientists have traditionally formalized the components of behavioral function using behavioral studies of normal and neurologically impaired individuals. As further reviewed in [77,93], the results of activation studies have challenged the classic models, as they have evidenced overlap between neural systems activated by tasks that share no known cognitive components. In agreement with a previous suggestion of systematically assigning labels that encompass the operations that each area performs [8], we propose to build a systematic ontology with a bottom-up perspective (starting from the biological substrate: the brain).

One challenge in the future will indeed be to integrate concepts and data from disparate brain science disciplines within a unified framework of brain biology [94]. Following up that perspective, building neurocognitive models (i.e., models combining cognitive and anatomical models) of normal functioning and pathology relies on the organization of the cognitive components into a single framework [16]. Such a theoretical position clearly implies that biological evidence should drive the revision of cognitive theories, provided that the new conceptualization derived from brain–behavior data has been robustly tested.

In addition to these theoretical considerations, one should also consider the clinical utility as a ‘quality marker’ for psychological ontologies. That is, a behavioral/cognitive taxonomy that is in agreement with brain organization should, in principle, also help researchers to understand, diagnose, or classify neurocognitive pathologies. Thus, in the future, assigning operation-function to brain regions should go hand in hand with the evolution of formal cognitive taxonomies, which in turn should benefit the understanding of neurocognitive pathology.

## Box 2. Real and Virtual Lesions: Strong but Complicated Evidence

Localized brain lesions and stimulation approaches producing ‘virtual’ lesions may be considered as providing the strongest evidence that the function of a brain region is causally related to a particular behavioral function, if damage to the region indeed disrupts the performance of the respective behavior. This level of interpretation is inaccessible for either activation studies or brain–behavior correlations, which are observational in nature and may not differentiate epiphenomena or spurious effects.

In turn, lesion–symptom associations are complicated by the high plasticity of the human brain (e.g., [95,96]), which results in substantial remodeling of circuitry as early as days after the insult. In addition, lesion locations are neither uniform nor random, but either follow a spatial structure determined by the vascular tree, in case of ischemic lesion, or mainly occur in surface structures, in the case of traumatic brain injury [49,97]. Furthermore, other factors may contribute to the observed pattern: for example, regions that appear to be structurally intact might have their function impaired by disconnection from, or damage to, an important ‘coworker’. As these regions are also unable to function despite being structurally intact, the perturbations ascribed to the damaged regions may be overestimated [97,98].

Noninvasive stimulation techniques offer an experimental approach to perturbation and avoid the limitations of neuropsychological lesion mapping such as plasticity (though there is evidence for short-term homeostatic effects), but their application is largely limited to surface structures [99,100]. Furthermore, the questionable focality of transcranial magnetic stimulation-induced currents [10] and influences of cortical geometry substantially impede the spatial specificity of brain stimulation. Finally, stimulation of densely connected regions will result in uncontrolled propagation of the pulse into spatially distant regions [5,6], inducing (uncontrolled) network effects. Thus, a well-controlled experiment based on a stimulation approach should include an examination of the propagation of the pulse, such as a compatible electroencephalographic recording, making this a technically challenging approach.

## Box 3. BrainMap and Neurosynth

BrainMap and Neurosynth are both collections of results of activation studies, consisting of reported coordinates and information about the experimental context. BrainMap [75,101] is based on manual encoding of spatial coordinates and behavioral conditions, according to an expert-defined ontology. That is, each reported set of coordinates is encoded with respect to the employed paradigm, the assessed contrast, and other aspects such as stimuli or required responses. This labeling is cross-validated by a second investigator, yielding a rigorous standard of labeling, resulting in rather slow growth and confinement to an *a priori* taxonomy.

Neurosynth [76], by contrast, is based on automated text-mining. First, coordinates are extracted from published neuroimaging articles using an automated parser, without distinction between different contrasts or experiments. Thus, one major difference between both databases is that BrainMap works on the experiment level (single contrast) and Neurosynth on the study level (complete paper). In the latter database, each article is then ‘tagged' with those terms that occur with high frequency in the abstract of the paper. This automated parsing has the advantage of not being limited by a predefined set of labels. By contrast, it comes with the drawback that the descriptions are solely based on the words used to describe the study by the authors. Consequently, the labels will summarize the conceptual terms that the researchers (or the reviewers) wanted to see addressed, not necessarily the behavioral function or process that was truly isolated.

Thus, both databases are based on current psychological ontologies and terminology but may provide slightly different behavioral information for a given brain region. For example, memory-related terms associated with the hippocampus in BrainMap would be ‘explicit memory’, while Neurosynth would provide terms such as ‘remember’, and ‘remembering’. While the former lacks semantic precision, the latter could be (spuriously) driven by the fact that many studies addressing ‘remember’ have focused on the medial temporal lobe. As region-of-interest studies are not excluded in Neurosynth, there is a potential for self-fulfilling prophecies. Hence, each approach has its own strengths and drawbacks, and deeper insights would likely result from their combination.

## Box 4. Brain Areas in Different Individuals

The brain’s spatial organization can be seen as a multiscale topographic layout. Mapping this organization into specialized regions that are, in turn, integrated into larger networks is addressed by various brain parcellation approaches. Several atlases are now available. Those atlases are averaged across population samples, representing critical maps for studying brain function and dysfunction. Nevertheless, this work is complicated by interindividual variability, particularly with respect to gyral folding patterns [102]. Furthermore, the organization of the cerebral cortex, rather than being seen as a set of clear-cut patches, could be seen as emerging from the crossing of different gradients of features organized along different axes [105].

Several methodological approaches have been proposed [in particular more recently in the framework of the Human Connectome Project (HCP) [84] and the UK Biobank [86]] to account for these factors that complicate the matching of areas in different individuals. One first line of improvement lies in the acquisition of MRI data at higher resolution, that can, for example, distinguish areas on opposite sides of sulcal banks. Additionally, registration based on surface features and examination of several gradients of features, such as myelin and resting-state signal, can further improve alignment of structural and functional units, respectively [102]. Also in contrast with traditional alignment, which is based on macro-anatomical features, hyperalignment (based on functional features, and hence in a functional space) [103] could significantly contribute to the study of functional units.

As aforementioned, both the HCP and the UK Biobank offer a range of psychometric data, but the HCP and the Individual Brain Charting Project also allow a characterization of activation across a range of behavioral paradigms. Thus, while BrainMap and Neurosynth can provide systematic activation-based functional profiling, averaged across individuals, the spatial mapping of the structure-function pattern can be further examined within datasets that allow high-evel alignment. These improvements have, for example, been used to identify a functional gradient lying horizontally from the posterior middle frontal gyrus to the crown of the precentral gyrus (area 55b), which is engaged when participants listen to stories [102].

## Highlights

While it is largely accepted that the brain is topographically organized into distinct areas that are integrated into networks, the unique contribution of each area to behavior is yet to be elucidated.

Diverse lines of research using different approaches have contributed to numerous behavioral associations for any brain area.

Emerging databases of task-based activation data offer the possibility of characterizing the engagement of brain regions across a broad range of experimental behavioral conditions.

New large samples of both imaging and phenotypical data provide an opportunity to complement the activation pattern by examining cross-subject associations between imaging-derived neurobiological markers and ecological behavioral characteristics.

## Outstanding Questions

To what extent do mental functions reflect regional specialization of individual brain regions, and the integration thereof into large-scale networks?

How can we integrate the different concepts and hypotheses regarding the behavioral functions of brain regions from different fields of research and different methodologies?

How could we characterize the function of a brain region by accounting for both segregation implemented in the local structure and integration supported by the connectivity pattern?

Can currently available cognitive ontologies be mapped onto the brain? That is, will we find that specific cognitive concepts can be mapped onto specific biological units?

How can we foster the integration and joint/complementary use of different methods, which all have their individual strengths and drawbacks?

When the systematic characterization of a brain region, based on data aggregation, has generated a new hypothesis, how can we combine different approaches to test this hypothesis in an unbiased fashion?

## Footnotes

i Resources https://project.inria.fr/IBC/data/

## Glossary

Ecological validity: an epistemological concept referring to the quality of the methods, materials, and settings of a study used to reproduce the examined real-life phenomena.
Epistemological: referring to the study of the nature and grounds of knowledge, especially regarding limits and validity.
Internal validity: a quality criterion for study designs that indicates the degree to which observed effects in some dependent variable can be assumed to be caused by the experimental manipulation; typically highest in randomized experiments and lowest, or absent, in correlative designs.
fMRI: can be used to measure hemodynamic changes related to neural activity during a particular mental task. Oxyhemoglobin and deoxyhemoglobin have different magnetic properties, which can be captured with an fMRI scanner. During mental activity, the ratio between oxyhemoglobin and deoxyhemoglobin is modified, allowing inference about the region of the brain in which neural activity changes during a particular mental task.
MRI: a technique of imaging body tissues (such as brain tissues) and physiological processes. It uses magnetic fields, radio waves, and field gradients. As different tissues have different magnetic properties, structural MRI can be used to generate an anatomical image of the brain that differentiates gray matter, white matter, and cerebro-spinal fluid.
Ontology: an explicit specification of the conceptual entities that are postulated by a theory. A formal ontology specifies the structure of the theory in terms of the elementary entities and their conceptual relationships.
Positron emission tomography (PET): a technique that measures physiological function by detecting blood flow and metabolism. It is based on the detection of radioactivity emitted after a radioactive tracer has been injected into the body. It can therefore be used to examine blood flow and oxygen consumption in different parts of the brain during a mental task.
Transcranial direct current stimulation: a technique of neurostimulation in which electrodes are placed on the head of a participant to induce currents. It changes neuron excitability by modifying the membrane polarization potential.
Transcranial magnetic stimulation (TMS): a technique used to stimulate the brain locally. A magnetic coil is placed close to the scalp (without physical contact with the head) to induce currents, which change the polarization of the neurons in the area under the coil. The neural effect of TMS depends on the frequency of the stimulation. It can be excitatory or inhibitory.
